# Maternal Psychological Problems During Pregnancy and Child Externalizing Problems: Moderated Mediation Model with Child Self-regulated Compliance and Polygenic Risk Scores for Aggression

**DOI:** 10.1007/s10578-021-01154-1

**Published:** 2021-03-20

**Authors:** Mannan Luo, Irene Pappa, Charlotte A. M. Cecil, Philip Jansen, Marinus H. van IJzendoorn, Rianne Kok

**Affiliations:** 1 Department of Psychology, Education and Child Studies, Erasmus University Rotterdam, Burg. Oudlaan 50, 3062 Rotterdam, PA Netherlands; 2 Generation R Study Group, Erasmus MC, University Medical Center Rotterdam, Rotterdam, Netherlands; 3 Department of Child and Adolescent Psychiatry/Psychology, Erasmus MC, University Medical Center Rotterdam, Rotterdam, Netherlands; 4 Department of Epidemiology, Erasmus MC, University Medical Center Rotterdam, Rotterdam, Netherlands; 5 Department of Complex Trait Genetics, Center for Neurogenomics and Cognitive Research, Amsterdam Neuroscience, Vrije Universiteit Amsterdam, Amsterdam, Netherlands; 6 Department of Clinical Genetics, Vrije Universiteit Medical Center, Amsterdam UMC, Amsterdam, Netherlands; 7 Department of Clinical, Education and Health Psychology, Faculty of Brain Sciences, UCL, University of London, London, UK

**Keywords:** Self-regulated compliance, Maternal psychological problems, Prenatal effect, Externalizing problems, Polygenic risk scores

## Abstract

A potential pathway underlying the association between prenatal exposure to maternal psychological problems and childhood externalizing problems is child self-regulation. This prospective study (*N* = 687) examined whether self-regulated compliance mediates the relation between maternal affective problems and hostility during pregnancy and childhood externalizing problems, and explored moderation by child polygenic risk scores for aggression and sex. Self-regulated compliance at age 3 was observed in mother–child interactions, and externalizing problems at age 6 were reported by mothers and teachers. Polygenic risk scores were calculated based on a genome-wide association study of aggressive behavior. Self-regulated compliance mediated the associations between maternal psychological problems and externalizing problems. Aggression PRS was associated with higher externalizing problems reported by mothers. No evidence was found of moderation by aggression PRS or sex. These findings support the hypothesis that maternal psychological problems during pregnancy might influence externalizing problems through early self-regulation, regardless of child genetic susceptibility or sex.

**Supplementary Information:**

The online version contains supplementary material available at 10.1007/s10578-021-01154-1.

## Introduction

Poor self-regulation in childhood has been implicated in a variety of maladaptive outcomes [1], including externalizing problems in childhood and adolescence [2, 3]. Child self-regulated compliance, as an important aspect of early self-regulation, involves the autonomous inhibition of inappropriate responses, and the regulation of attention and behavior [4, 5]. In contrast to externally regulated compliance, self-regulated compliance refers to the child’s willingness to follow initial directives even in the absence of sustained parental control [6].

**Fig. 1:**
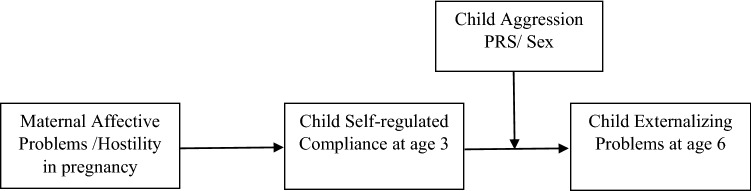
Conceptual model of moderated mediation

Despite prenatal exposure to maternal affective problems (i.e., depression and anxiety) has been linked to both childhood regulatory difficulties [7–10] and externalizing problems [11–16], little research to date has investigated how child self-regulation may help explain externalizing outcomes associated with maternal psychological problems in pregnancy. As such, the purpose of the present study was to examine whether child self-regulated compliance mediates the association between prenatal exposure to maternal psychological problems and later externalizing problems, as well as child characteristics (i.e., genetic risk factors, sex) that may moderate these associations (see Fig. 1).

Previous studies have largely investigated the effect of postnatal maternal factors, such as parenting behavior on child self-regulated compliance [17, 18]. However, a few studies on intra-uterine mechanisms have identified the prenatal period as a starting point for the development of children’s self-regulation [19, 20]. Maternal affective problems during pregnancy place the fetus at risk of greater exposure to maternal cortisol which may affect the developing fetal neurobiological system (e.g., hypothalamic pituitary axis (HPA) and prefrontal cortex (PFC)) [21, 22]. Specifically, dysregulated HPA functioning, which can exaggerate and prolong stress responses, has been linked to difficulties in effortful control and behavioral regulation in children [23, 24]. In addition, the PFC is also implicated in behavioral self-regulation processes, such as executive attention and inhibitory control [25, 26]. As a consequence, these children might show more emotional and/or behavioral problems later in life.

There is an increasing body of literature illustrating a similar role for other psychological problems during pregnancy. Child regulation and externalizing problems are also affected by maternal hostility [27, 28], which is characterized by outward-directed anger, irritability and urges to hit or injure others. Field et al. [29, 30] found that high levels of prenatal maternal hostility are associated with elevated levels of cortisol and adrenaline, in both mothers and neonates, that may affect children’s development in a similar way to prenatal affective symptoms. Although these studies provide indirect support for the hypothesis that self-regulated compliance mediates the association between prenatal exposure to maternal psychological problems and later externalizing problems of the child, this hypothes is awaits direct empirical testing.

Besides our focus on the mediating effect of children’s self-regulation, we also considered the moderating roles of individual characteristics of the child, such as genetic predisposition to aggression and sex. Given previous research showing that complex behavioral traits are influenced by common genome-wide genetic variants with small effects, we follow a polygenic approach by using data from the largest genome-wide association study (GWAS) to date of aggression in childhood [31, 32]. This approach increases the prediction accuracy of genetic influence compared to a candidate gene approach [33]. In the present study, for the first time, we incorporated genetic predisposition in our conceptual model for externalizing problems, hypothesizing that young children with higher polygenic risk score (PRS) for aggression and lower levels of self-regulation would have more risk to develop externalizing problems later in childhood.

Child sex could also influence the relation between self-regulated compliance and externalizing problems. There is some evidence that boys tend to display more difficulties with self-regulation [18, 34, 35], as well as more externalizing problems compared to girls [36]. However, previous literature remains inconsistent; a few studies reported that low levels of self-regulation were associated with more behavior problems among boys compared to girls [37, 38], whereas others indicated stronger associations for girls but not for boys [39] or no sex differences [40].

Although studies into the relations among maternal psychological problems, children’s self-regulation and externalizing problems have accumulated, the research is limited in two main ways. First, studies have assessed maternal psychological problems postnatally or cross-sectional to child self-regulation or externalizing problems, which cannot preclude a reverse association. Second, most studies showing an association between child self-regulation and externalizing problems have not taken into account genetic influences. Self-regulation and externalizing problems both are heritable traits [41, 42] and shared genetic effects could underlie their observed association. Furthermore, genetic factors may interact with self-regulatory ability to increase the risk of externalizing problems. To address these gaps, the present study used prospective data from the Generation R Study, a large-scale population-based study with multi-method and multi-informant measurements. We examine whether child self-regulated compliance mediates the association between prenatal psychological problems and child externalizing problems, accounting for the potential moderating effect of child genetic susceptibility and sex. We hypothesized that maternal psychological problems in pregnancy (i.e., both affective problems and hostility) are associated with lower levels of child self-regulated compliance at age 3, which in turn predict higher levels of mother- and teacher-reported externalizing problems at age 6. We also hypothesized that the association between self-regulated compliance and externalizing problems is stronger for children with a genetic predisposition to aggression (i.e., higher PRS of aggression). Because of inconsistent findings for sex effects on self-regulation and externalizing problems, we did not have a specific hypothesis about the moderating effect of child sex.

## Method

### Settings

The current study was embedded within the Generation R Study, a prospective population-based cohort study investigating growth, development, and health from fetal life onwards in Rotterdam, the Netherlands. Detailed measurements were obtained in a subgroup of children of Dutch national origin, meaning that the children, two parents and four grandparents were all born in the Netherlands [43, 44]. Further eligibility criteria for participation in this study were enrollment before a gestational age of 25 weeks and a delivery date between February 2003 and August 2005. The study was conducted in accordance with the ethical standards for human experimentation established by the Declaration of Helsinki and was approved by the Medical Ethical Committee of the Erasmus MC, University Medical Centre Rotterdam. More information about the study design can be found elsewhere [45, 46].

### Participants

The current study incorporates self-reported maternal psychological problems during pregnancy, observed child compliance at age 3 years, and mother- and teacher-reported externalizing behavior at child’s age 6 years. In the laboratory visit around 3 years of age (*M* = 3.13, *SD* = 0.13), 852 children and their primary caregiver participated. Of these, 31 children were excluded because of technical or procedural difficulties during the two compliance tasks. Thus, for 821 children data on compliance was available (at least one out of two tasks). Within this group, 21 mothers participated in the 3 year visit twice, with twins or siblings. One child of each sibling pair was randomly excluded to avoid bias due to paired data. Of the remaining 800 mother–child dyads, information on affective problems and hostility during pregnancy was available for 750 mothers. Of this group, children without data on externalizing problems were excluded (Mother reports: *n* = 63; Teacher reports: *n* = 290). This resulted in two final samples for the mediation analyses, *N* = 687 for analyses predicting maternal-reported externalizing problems and *N* = 460 for analyses predicting teacher-reported externalizing problems. Additionally, moderated mediation analyses were performed to assess a potential moderating effect of PRS for aggression in subsamples with genetic data available [31], in 522 of the 687 children (mother reports) and 353 of the 460 children (teacher reports).

For the nonresponse analyses, we compared child and maternal characteristics of mother–child dyads included in the analyses (*n* = 687) with those excluded because of missing data on mother report externalizing problems (*n* = 113). Excluded children showed less self-regulated compliance (*M* = − 0.21, *SD* = 0.93) at age 3 than children included in the analyses (*M* = 0.03, *SD* = 0.87), Cohen's *d* = 0.27, *t*(798) = − 2.69, *p* = 0.01. Excluded children were also more likely to be younger at the assessment of externalizing problems, Cohen's *d* = 0.36, *t*(728) = − 2.45, *p* = 0.01, compared to included children. Nonresponse analyses also comparing the subsample of teacher-reported externalizing problems, excluded mother–child dyads (*n* = 340) did not significantly differ from those included in the analyses (*n* = 460) on background variables, maternal psychological problems during pregnancy and child self-regulated compliance.

### Measures

#### Maternal Affective Problems and Hostility During Pregnancy

The Dutch version [47] of the Brief Symptom Inventory (BSI) [48] was administered between 20 and 25 weeks of pregnancy (*M* = 20.6, *SD* = 1.2) to measure maternal affective problems and hostility. The BSI is a validated self-report questionnaire with 53 items on a 5-point scale, ranging from 0 = ‘not at all’ to 4 = ‘extremely’. The items of the BSI cover nine subscales of psychological problems occurring in the preceding 7 days. For the current study, we used the depression, anxiety and hostility subscales. Maternal affective problems represent a composite of the Depression subscale (e.g. “Feeling suicidal” and “Having no interest in anything”) and the Anxiety subscale (e.g. “Nervousness or shakiness inside”, “Suddenly scared for no reason”), each containing 6 items. Maternal hostility was based on the Hostility subscale, which consists of 5 items such as “I have an urge to hit, injure or cause pain to others” and “I often get involved in arguments”. Total scores for each scale were calculated by summing the item scores and then dividing by the number of endorsed items, with a maximum of one missing item allowed as recommended in the BSI manual [47]. The internal consistency (Cronbach’s *α*) in this sample was *α* = 0.88 for Affective problems and *α* = 0.75 for Hostility during pregnancy. Scale scores were *z*-standardized to facilitate interpretation.

#### Self-regulated Compliance

Self-regulated compliance was assessed at 3 years in a sequence of two disciplinary contexts (*Don’t task*; 2 min each). During the *Don’t task ‘no touching’* context, the parent prohibited the child to touch or play with a set of attractive toys that were displayed before the child; during the *Don’t task ‘least-attractive toy’*, the parent allowed the child to play with an unattractive teddy bear, but still prohibited the child to touch or play with the attractive toys. Child behavior was coded every 20 s using a coding system based on Kochanska and Aksan [49] and Kuczynski, Kochanska, Radke-Yarrow and Girnius-Brown [50]. The predominant behavior of the child in the twelve 20 s segments was coded in four mutually exclusive categories. Committed compliance was coded if the child appeared to embrace the parental agenda wholeheartedly, made no attempt to touch or play with the toys, and needed no prompting by the parent. Situational compliance was coded when the child needed regular prompting and/or showed difficulty in complying. Passive noncompliance was coded if the child ignored the mother’s request. Resistant noncompliance was coded when a child actively resisted the mother, that is, protesting or whining [18].

The data were independently coded by six coders who were extensively trained and regularly supervised. For the *Don’t task (no touching)*, the inter-coder reliability (intra-class correlation coefficients, ICC) among the four coders after the training was 0.74 on average (*n* = 20), and 0.63 at the end of the coding process (*n* = 33), with an ICC of 0.68 over the total set (*n* = 53). For the *Don’t task (least-attractive toy),* the ICC between the two coders directly after the training was 0.81 on average (*n* = 20) and 0.84 at the end of the coding process (*n* = 33), with an ICC of 0.87 over the total set (*n* = 53) [18].

CATPCA (Categorical Principal Components Analysis) [51] was used to investigate the correlation structure of the data. A one-dimensional structure explained 48% of variance for *Don’t task (no touching*) and 50% of variance for *Don’t task (least-attractive toy).* The dimension was labeled *Self-regulated Compliance*. The correlation between two factor scores across tasks was *r* (788) = 0.43, *p* < 0.001. To increase the reliability and avoid collinearity, an overall score for self-regulated compliance was created by averaging the two factor scores, with higher scores representing more self-regulated compliance across the two Don’t tasks.

#### Externalizing Problems

Mothers and teachers reported child externalizing problems at approximately 6 years of age (mother-report: *M* = 5.87, *SD* = 0.19; teacher-report *M* = 6.44 years, *SD* = 1.06) on the Child Behavior Checklist (CBCL/1.5–5) [52] and the Teacher Report Form (TRF/6–18) [53]. All items on both measures were rated on a 3-point scale (0 = not true, 1 = somewhat true or sometimes true, 2 = very true or often true) on the basis of the child's behaviors in the preceding two months. For each subscale, items were summed, with higher scores representing more problem behavior. Good reliability and validity have been reported for the CBCL and the TRF [52, 53].

We used the 24-item Externalizing broadband scale of the CBCL comprising Attention problems and Aggressive behavior. The Attention Problems scale includes five items such as “Can't sit still, restless, or hyperactive,” and “Wanders away.” The Aggressive Behavior scale consists of 19 items such as “Hits others,” and “Demands must be met immediately.” From the TRF, we used the 32-item Externalizing broadband scale comprising aggressive and rule-breaking behavior. The Aggressive Behavior scale consists of 20 items such as “Physically attacks people” and “Cruelty, bullying or meanness to others.” The Rule Breaking Behavior scale includes 12 item such as “Lies, cheats” and “Breaks rules.” The internal consistencies for the Externalizing problem scores (Mother report, Cronbach’s *α* = 0.89; Teacher report, Cronbach’s* α* = 0.91) were very good in the current study. The externalizing scores were *z*-standardized to facilitate interpretation.

#### Polygenic Risk Scores for Child Aggression

PRSs were calculated on imputed genotype data using publicly available GWAS results for childhood aggression (*N* = 18,988; age range: 3–15 years) [31], in which the Generation R data was included. To ensure independence of the discovery and target sample [32], we repeated the meta-analysis with GWAS results of the other 8 cohorts after exclusion of the Generation R cohort (sample size after exclusion = 16,778). Genotype data collection and subsequent processing procedures for Generation R have been described previously [54]. Additional quality control and imputation are described in the Supplement 1.

We calculated PRSs using PRSice-2 [55], based on several *p*-value thresholds (*p* < 0.01, 0.1, 1) for inclusions of single nucleotide polymorphisms (SNPs) in the score. SNPs were clumped according to linkage disequilibrium (LD) to obtain the most significant SNP per LD block (250-kb window, *r*^2^ < 0.1). We tested multiple thresholds to find the optimal threshold that has the strongest association with externalizing problems. PRS were standardized to a mean of 0 and a SD of 1 for interpretational purposes.

#### Covariates

Child sex, age at the assessment of outcomes and maternal educational level were selected based on previous literature that found associations between these covariates and main variables. Child sex was included because self-regulated compliance and externalizing problems may differ between boys and girls [18, 34]. Information on sex was obtained from community midwife and hospital registries at birth. Maternal educational level was dichotomized into “higher” (at least higher vocational training or a bachelor’s degree) versus “lower” (primary or secondary education). Of note, we did not adjust for genetic principal components when examining the role of aggression PRS, because of the relatively homogenous population structure in the Dutch cohort study.

### Statistical Analyses

Associations among the main study variables were explored using Pearson’s correlations. All subsequent analyses of the study were adjusted for covariates described in the previous section. Because the distributions of maternal prenatal psychological problems and child externalizing problems were skewed, the bootstrap resampling method was applied [56]. The bias-corrected bootstrap 95% confidence interval (CI) with 5000 resamples estimates the size and significance of the indirect effect. Following procedures proposed by Hayes [57], data analysis was conducted using the Process macro for SPSS. Model 4 was used to test the mediating effect of self-regulated compliance. Model 14 was used to test moderated mediation effects.

First, we examined whether self-regulated compliance at age 3 years mediated the relation between prenatal maternal psychological problems and child externalizing problems at age 6 years using Model 4. We tested four separate models, including either prenatal maternal affective or hostility problems as two separate predictors, and either teacher or mother reports of child externalizing problems as two separate outcomes. If a statistically significant mediation was detected, we then conducted moderated mediation analyses using Model 14. Child aggression PRS or sex were entered respectively as moderators of the association between self-regulated compliance and externalizing problems. Finally, we calculated the index of moderated mediation, which indicates whether the mediating effects of prenatal maternal psychological problems on child externalizing problems through self-regulated compliance are similar across child aggression PRS or sex.

In addition to the main analyses described above, we performed sensitivity analyses to test the robustness of our findings. For sensitivity analysis, the mediation models were additionally adjusted for concurrent maternal psychological problems assessed with self-regulated compliance at child age 3, in order to obtain independent prenatal effects.

## Results

### Mean Differences and Bivariate Correlations Among Main Variables

The analytic sample of 687 children comprised 50.2% boys, 66.7% of mothers finished higher education. Externalizing problems were assessed at the mean age of 5.87 years (*SD* = 0.19) on mother reports, at the mean age of 6.44 years (*SD* = 1.06) on teacher reports. Mothers of boys reported more hostility during pregnancy (*M* = 0.22, *SD* = 0.34) than mothers of girls (*M* = 0.16, *SD* = 0.22), Cohen's *d* = 0.21, *t*(685) = − 2.70, *p* = 0.001. Boys were less compliant (*M* = − 0.71, *SD* = 0.17) than girls (*M* = 0.76, *SD* = 0.14), Cohen's *d* = 0.31, *t*(685) = 4.09, *p* < 0.001. Mothers reported more externalizing problems in boys (*M* = 7.51, *SD* = 6.40) than in girls (*M* = 5.47, *SD* = 5.06), Cohen's *d* = 0.35, *t*(685) = − 4.63, *p* < 0.001. Teachers also reported more externalizing problems in boys (*M* = 2.95, *SD* = 5.41) than in girls (*M* = 1.05, *SD* = 3.11), Cohen's *d* = 0.43, *t*(458) = − 4.60, *p* < 0.001.

As shown in Table 1, bivariate correlations indicated that children who were prenatally exposed to higher levels of maternal affective problems and hostility showed less self-regulated compliance, and more externalizing problems on mother reports, but not teacher reports, although consistent in the direction of associations. Moreover, children with lower levels of self-regulated compliance exhibited more externalizing problems on both mother and teacher reports. Mother- and teacher- reported externalizing problems were moderately correlated. We also found that higher levels of aggression PRS was significantly correlated with more externalizing problems as rated by mothers and teachers. There was no significant correlation between aggression PRS of the child and prenatal affective problems or hostility of the mother. Additionally, we found that girls showed more self-regulated compliance and less externalizing problems than boys.

**Table 1:** Descriptive statistics and correlations of main variables (*N* = 687)

	*M*	*SD*	1	2	3	4	5	6
1 Maternal affective problems during pregnancy	0.14	0.27	–					
2 Maternal hostility during pregnancy	0.19	0.29	0.66**	–				
3 Self-regulated compliance	0.74	0.16	− 0.10**	− 0.10**				
4 Mother-reported externalizing problems	6.48	5.86	0.16**	0.11**	− 0.16**	–		
5 Teacher-reported externalizing problems ^a^	2.04	4.59	0.07	0.08	− 0.14**	0.36**^b^	–	
6 Aggression PRS ^c^	− 0.02	0.01	0.04	0.04	− 0.05	0.12**	0.11*^d^	–
7 Child sex, boys	–	–	0.07	0.10**	− 0.15**	0.17**	0.22**	0.08

^a^*n* = 460

^b^*n* = 427

^c^*n* = 522

^d^*n* = 353

^*^p < .05

**p < .01

### Mediating Role of Self-regulated Compliance

#### Maternal Affective Problems During Pregnancy and Externalizing Problems

**Fig. 2:**
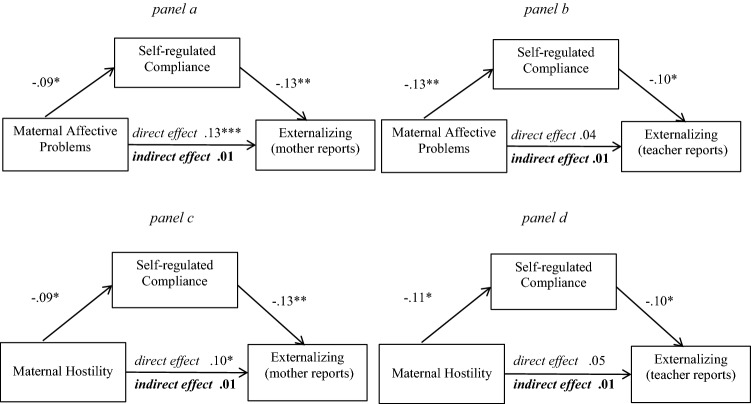
Four separate mediation models for the effects of maternal affective problems/hostility on mother- and teacher-reported externalizing problems. Coefficients are standardized estimates; Significant confidence intervals are in bold. *p < .05, **p < .01, ***p < .001

As shown in Fig. 2 panel a, self-regulated compliance at age 3 significantly mediated the association between maternal affective problems during pregnancy and mother reported externalizing problems of the child at age 6 (*β* = 0.01, SE = 0.01, 95% CI [0.003, 0.029]). The direct effect of maternal affective problems on mother-reported externalizing behaviors was significant (*β* = 0.13, SE = 0.04, 95% CI [0.059, 0.205], *p* < 0.001). Specifically, the higher levels of maternal affective problems during pregnancy (*β* = − 0.09, SE = 0.04, 95% CI [− 0.166, − 0.018], *p* = 0.02) were associated with less self-regulated compliance in children, which, in turn, predicted more externalizing problems as rated by mothers (*β* = − 0.13, SE = 0.04, 95% CI [− 0.198, − 0.051], *p* = 0.001). Additionally, the mediating effect of self-regulated compliance on the relation between maternal affective problems during pregnancy and teacher reported externalizing problems at age 6 (Fig. 2, panel b), was significant, *β* = 0.01, SE = 0.01, 95% CI [0.002, 0.037], but the direct effect for teacher reports was not, *β* = 0.04, SE = 0.05, 95% CI [− 0.052, 0.126], *p* = 0.41. Coefficients for the full model are presented in Table 2. Sensitivity analyses with the additional adjustment for co-occurring maternal affective problems at child age 3 showed similar results, with the two differences that the direct effect of prenatal exposure to maternal affective problems on externalizing problems was non‐significant for mother reports (see Supplement Table S1).

**Table 2:** Mediation models: self-regulated compliance as mediator of maternal psychological problems – child externalizing problems associations

Predictors	Outcomes: externalizing problems					
	Mother reports (*N* = 687)			Teacher reports (*N* = 460)		
	*b* (SE)	95% CI	*β*	*b* (SE)	95% CI	*β*
Maternal affective problems during pregnancy (direct effect)	2.90 (0.82)	**1.29, 4.50**	0.13	0.56 (0.68)	− 0.78, 1.89	0.04
Indirect effect via self-regulated compliance	0.25 (0.14)	**0.05, 0.63**	0.01	0.20 (0.13)	**0.03, 0.56**	0.01
Maternal hostility during pregnancy (direct effect)	1.69 (0.76)	**0.20, 3.19**	0.08	0.66 (0.60)	− 0.52, 1.84	0.05
Indirect effect via self-regulated compliance	0.23 (0.14)	**0.04, 0.60**	0.01	0.15 (0.11)	**0.02, 0.45**	0.01

All covariates are included in the model as described in Methods section

Paths significant as indicated by the confidence intervals are presented in bold

*b* unstandardized coefficients with 95% confidence intervals, *SE* standardized error, *β* standardized coefficients

#### Maternal Hostility During Pregnancy and Externalizing Problems

Parallel findings emerged for maternal hostility during pregnancy, as shown in Fig. 2 panel c and d respectively. The results indicated a mediating effect of self-regulated compliance on the association between maternal hostility during pregnancy and mother-reported externalizing problems (*β* = 0.01, SE = 0.01, 95% CI [0.002, 0.030]). The direct effect of maternal hostility on mother-reported externalizing behaviors was significant (*β* = 0.10, SE = 0.04, 95% CI [0.010, 0.158], *p* = 0.03). Specifically, the higher levels of maternal hostility during pregnancy (*β* = − 0.09, SE = 0.04, 95% CI [− 0.161, − 0.011], *p* = 0.03) was associated with less self-regulated compliance in children, which, in turn, predicted more externalizing problems at age 6 as rated by mothers (*β* = − 0.13, SE = 0.04, 95% CI − 0.204, − 0.055], *p* = 0.001). Additionally, the mediating effect of self-regulated compliance on the relation between maternal hostility during pregnancy and teacher reported externalizing problems at age 6 (see panel d), was significant (*β* = 0.01, SE = 0.01, 95% CI [0.001, 0.034]), but the direct effect for teacher reports was not (*β* = 0.05, SE = 0.05, 95% CI [− 0.039, 0.139], *p* = 0.27). When we additionally adjusted for maternal hostility at child age 3, the magnitude of the associations remained similar to the main analyses, although the mediating effects were no longer significant (Supplement Table S1).

### Moderating Effect of Child Aggression PRS or Sex

As shown in Table 3, the index of moderated mediation indicated that child aggression PRS did not moderate the mediating effect of self-regulated compliance in the relation between maternal affective problems during pregnancy and child externalizing problems as rated by mothers or teachers. Child aggression PRS significantly predicted mother-reported externalizing problems (*β* = 0.10, SE = 0.04, 95% CI [0.011, 0.178], *p* = 0.03*).* The PRS explained about 1% of the variance in mother-reported externalizing problems (*R*^2^ = 0.01, see Supplement Table S2). While controlling for child aggression PRS, the effect of self-regulated compliance on externalizing problems remained significant. Similar results were found in the model with maternal hostility during pregnancy. Aggression PRS also did not moderate the mediating effect in the link examined but this PRS did predict mother-reported externalizing problems.

**Table 3:** Moderated mediation analyses: maternal psychological problems during pregnancy predicting child externalizing problems, mediated by self-regulated compliance, moderated by aggression PRS

Predictors	Outcome: externalizing problems			
	Mother reports (*N* = 522)		Teacher reports (*N* = 353)	
	*β* (SE)	95% CI	*β* (SE)	95% CI
Maternal affective problems during pregnancy	0.12 (0.04)	**0.032, 0.202**	0.01 (0.05)	− 0.094, 0.109
Self-regulated compliance	− 0.10 (0.04)	− **0.186, − 0.017**	− 0.07 (0.05)	− 0.173, 0.032
Aggression PRS	0.10 (0.04)	**0.011, 0.178**	0.06 (0.05)	− 0.045, 0.157
Self-regulated compliance*aggression PRS	− 0.05 (0.04)	− 0.133, 0.030	0.03 (0.05)	− 0.073, 0.125
Child sex	0.32 (0.09)	**0.154, 0.492**	0.37 (0.10)	**0.168, 0.579**
Index of moderated mediation	0.01 (0.01)	− 0.001, 0.021	− 0.003 (0.01)	− 0.022, 0.006
Maternal hostility during pregnancy	0.06 (0.04)	− 0.025, 0.145	0.01 (0.05)	− 0.112, 0.091
Self-regulated compliance	− 0.11 (0.04)	− **0.192, **− **0.022**	− 0.07 (0.05)	− 0.174, 0.030
Aggression PRS	0.10 (0.04)	**0.012, 0.180**	0.06 (0.05)	− 0.045, 0.158
Self-regulated compliance*aggression PRS	− 0.05 (0.04)	− 0.130, 0.035	0.03 (0.05)	− 0.072, 0.125
Child sex	0.33 (0.09)	**0.159, 0.498**	0.38 (0.11)	**0.170, 0.581**
Index of moderated mediation	0.004 (0.01)	− 0.001, 0.019	− 0.003 (0.01)	− 0.020, 0.004

All covariates are included in the model as described in Methods section

Paths significant as indicated by the confidence intervals are presented in bold

*β* standardized coefficient, *SE* standardized error

As shown in Table 4, the index of moderated mediation indicated that child sex did not moderate the mediating effect of self-regulated compliance on the relation between maternal affective problems during pregnancy and externalizing problems as rated by both mothers and teachers. Self-regulated compliance did not interact with child sex to predict externalizing problems. Similar results were found in the model with maternal hostility during pregnancy. Coefficients for the full models are presented in Tables 3 and 4.

**Table 4:** Moderated mediation analyses: maternal psychological problems during pregnancy predicting child externalizing problems, mediated by self-regulated compliance, moderated by child sex

Predictors	Outcome: externalizing problems			
	Mother reports ( *N* = 687)		Teacher reports ( *N* = 460)	
	*β* (SE)	95% CI	*β* (SE)	95% CI
Maternal affective problems during pregnancy	0.13 (0.04)	**0.061, 0.207**	0.04 (0.05)	− 0.052, 0.125
Self-regulated compliance	− 0.12 (0.04)	− **0.195, **− **0.046**	− 0.09 (0.05)	− 0.180, 0.003
Child sex	0.29 (0.08)	**0.145, 0.439**	0.36 (0.09)	**0.182, 0.538**
Self-regulated compliance * Child sex	− 0.04 (0.08)	− 0.186, 0.112	− 0.15 (0.09)	− 0.321, 0.041
Index of moderated mediation	0.004 (0.01)	− 0.011, 0.028	0.02 (0.02)	− 0.001, 0.062
Maternal hostility during pregnancy	0.08 (0.04)	**0.009, 0.157**	0.05 (0.05)	− 0.044, 0.134
Self-regulated compliance	− 0.13 (0.04)	− **0.202, **− **0.051**	− 0.09 (0.05)	− 0.180, 0.002
Child sex	0.29 (0.08)	**0.142, 0.439**	0.36 (0.09)	**0.179, 0.536**
Self-regulated compliance * Child sex	− 0.03 (0.08)	− 0.184, 0.116	− 0.14 (0.09)	− 0.320, 0.042
Index of moderated mediation	0.003 (0.01)	− 0.011, 0.027	0.02 (0.01)	0.000, 0.060

All covariates are included in the model as described in Methods section

Paths significant as indicated by the confidence intervals are presented in bold

*β* standardized coefficient, *SE* standardized error

## Discussion

In this population-based prospective study, we examined the potential mediating role of child self-regulated compliance in the association between maternal psychological problems during pregnancy and later externalizing problems in children. We found that children of mothers struggling prenatally with psychological problems showed lower levels of self-regulated compliance at age 3, which in turn were related to higher levels of externalizing problems at age 6. The observed mediation effects were consistent across subtypes of maternal psychological problems and across informants of child externalizing problems. In addition, we used a polygenic risk score approach to disentangle potential genetic confounding in the association between early self-regulation compliance and later externalizing problems. We found that both self-regulated compliance and aggression PRS contribute independently to the development of externalizing problems, with no significant interaction effect. While boys have lower levels of self-regulated compliance and higher levels of externalizing problems compared to girls, there were no consistent interaction effects of self-regulated compliance and sex.

Our study indicates that maternal psychological problems during pregnancy are related to mother-reported, but not to teacher-reported, externalizing problems. This is consistent with research indicating prenatal exposure to elevated maternal psychological problems increases children’s risk of behavior problems [15, 16]. Several explanations may underlie this finding. First, these cross-informant discrepancies could be the result of different perspectives of the reporters or the result of situation specificity of behavior (e.g., at home vs. at school), which represent reliable and meaningful variation [58]. For example, Loomans*, *et al*.* [59] found that higher levels of maternal anxiety during pregnancy were associated with more externalizing problems at age 5 years (e.g., hyperactivity/inattention problems, conduct problems) as rated by mothers. When child behavior was rated by teachers, children prenatally exposed to higher levels of maternal anxiety showed more peer-relationship problems. On the other hand, it can be argued that the stronger association found for mother reports is due to rater bias [60], as depressive or hostile mothers may perceive their children’s behavior to be more problematic, when these prenatal symptoms persist into the postnatal period. It is also possible that our analyses on teacher reports were underpowered to detect a potential association, particularly given its smaller sample size and more restricted variation (*M* = 2.04, *SD* = 4.59) compared with mother reports (*M* = 6.48, *SD* = 5.86).

Previous research has largely focused on the importance of parenting behavior and family environment as a mechanism linking maternal affective problems to externalizing problems in childhood [61–63]. The current study extended past work by identifying another possible mechanism through which maternal affective problems affect subsequent externalizing problems: by impairing offspring self-regulated compliance in early childhood. The child with self-regulated compliance embraces the parental agenda and social standards, and thus experiences compliance as autonomous [4]. Ultimately, self-regulated compliance may lead to voluntary, thoughtful, adaptive, and effective regulatory capacities, even in the absence of external monitors, which may be particularly crucial during the transition to school. Our findings suggest that self-regulated compliance at age 3 can mediate the risk from prenatal exposure to maternal affective problems to children's externalizing outcomes 6 years.

Additionally, our study examined the prenatal effect of maternal hostility on later externalizing problems in offspring. Although maternal hostility during postnatal periods has previously been positively linked to infant regulatory processes [28] and behavioral problems in childhood [64], it was unclear whether and how prenatal exposure to maternal hostility affects child outcomes. We assumed that high levels of prenatal hostility may also reflect mothers’ own regulatory difficulties to cope with stressors. Maternal hostility during pregnancy may place the fetus at high risk of being exposed to elevated maternal stress hormones, such as cortisol [65], eventually resulting in compromised self-regulatory processes of the offspring. In a recent meta-analysis, almost 1.5 to 2 times higher risk of behavioral problems was found in children of mothers reported higher levels of affective problems during pregnancy, independent of postnatal symptoms [10]. Previous research has demonstrated that the persistence of prenatal effects can be explained by both genetic and environmental factors [18, 66]. For example, genetic factors that influence maternal hostility are inherited by offspring, thereby conferring risk on the development of externalizing problems in offspring. In addition, these genetic loading for maternal hostility may be indirectly reflected in the parenting behavior (e.g., harsh parenting) in the postnatal period, affecting child externalizing problems. Our findings are consistent with the hypothesis that prenatal exposures to both affective problems and hostility can have persisting effects on future problem behaviors in children via impaired self-regulation.

It is important to note that our study indicates significant, yet small, mediating effects of self-regulated compliance between prenatal maternal psychological problems and externalizing problems in childhood. Since the assessments of maternal psychological problems (20 weeks pregnancy), child self-regulated compliance (age 3) and externalizing problems (age 6) were conducted over a large span of time, we expect to see that the magnitude of effects decreases. Over this large time span, protective factors, such as social support, can appear and might buffer the negative impact [67] of prenatal maternal psychological problems. It is also possible that if prenatal psychological symptoms persist or even increase during the postnatal period, they may negatively affect mother–child interactions, which in turn could compromise further the early development of self-regulation. However, when maternal affective problems and hostility at child age 3 were controlled for separately in our sensitivity analyses, the effects of maternal psychological problems during pregnancy on child self-regulated compliance were not explained by corresponding postnatal problems. Finally, the nature of our low-risk population sample probably may have led to an underestimate of the observed effects. Thus, stronger mediating effects may be present when examining maternal psychological problems and child externalizing problems in clinical samples. Our mediation model has confirmed previous findings that lower levels of self-regulated compliance during toddlerhood predict more externalizing problems in school age [68, 69]. Self-regulation and child externalizing problems are both known to have a heritable component [41, 42]. Twin studies indicated that the relation between self-regulation and externalizing problems is partially driven by shared genetic factors [69, 70]; however, it has not been examined yet to which extent child self-regulatory difficulties and externalizing problems are influenced by common genetic variants across the entire genome. Given our earlier work of the PRS [71], we expected to observe an association between the aggression PRS and externalizing problems, as the genetic overlap between psychiatric traits is substantial [72]. This study is the first, to our knowledge, to investigate the contribution of early self-regulation to children's externalizing problems while taking into account genetic predispositions.

Contrary to our hypothesis, child aggression PRS did not moderate the association between self-regulated compliance and externalizing problems. Despite the relatively large sample size in the current study, it is still possible that we were underpowered to detect a significant interaction effect. Consistent with previous studies [73], aggression PRS only explained a small percentage of the variance in externalizing problems. The low explained variance could be the result of the limited sample size of aggression GWAS, which reduced the power to accurately detect genetic risk variants. We suggest that larger GWAS are needed to be able to construct polygenic risk scores with better predictive ability of children’s externalizing problems. Although the PRS may not be especially predictive of externalizing problems at the population level, our results suggest that both self-regulated compliance and aggression PRS have an independent impact on externalizing problems of the child.

### Strengths and Limitations

The present study has several strengths including the relatively large sample and the prospective design which limits the risk of reversed causality. Another strength of this study is the use of independent assessments (i.e., self-reports of maternal psychological problems, independent observational measurement of self-regulated compliance, and multi-informant reports on externalizing problems), which accounts for the potential influence of shared method variance and thereby further enhances the validity of our findings. Moreover, using polygenic risk scores, the present study demonstrates the importance of early self-regulated compliance for externalizing problems, independent of the effect of potentially shared genetic risks.

Yet, the results of the present study should be interpreted taking into account its limitations. First, despite using prospective data, causal conclusions cannot be drawn, given that transactional relations may also exist between child self-regulation and externalizing behavior [74]. Longitudinal or experimental studies are needed to confirm the causal directions. Second, it is difficult to disentangle whether the discrepancies between mother and teacher reports of externalizing problem are due to sample size differences, rater bias (mother reports) and/or the specificity of rating context. Third, our data do not allow us to determine which mechanisms, e.g. intrauterine programming, additive effects of maternal symptoms in prenatal and postnatal periods, spillover effects on mother–child interaction, may explain the observed association between maternal prenatal exposure and child self-regulation. Fourth, our study used a self-reported measure to assess maternal psychological problems during one week around 20 weeks of pregnancy, which can bias our results and fail to capture the chronic effects of these problems across the whole pregnancy. Further research is needed to complement these findings using clinician-administered ratings and data at multiple time points during pregnancy. Fifth, excluded children were younger and less self-regulated than those included. The absence of these participants in our study may affect the generalizability of our results. Last, although the adjustment for aggression PRS does partly account for shared genetic liability for aggression in both hostile mothers and offspring, we cannot fully disentangle the maternal prenatal versus genetic effects as we only used the child’s genetic information.

## Summary

Overall, our study provides evidence that maternal psychological problems (i.e., affective problems and hostility) during pregnancy might influence the development of children’s subsequent externalizing behavior by affecting their self-regulated compliance during early childhood. Furthermore, the study provides evidence that the mediating effect of self-regulation did not depend on child’s genetic predisposition to aggression and sex. In this population-based sample, both self-regulated compliance in early childhood and aggression PRS were uniquely associated with externalizing problems in later childhood. All in all, these findings illustrate the importance of both prenatal environment factors (i.e., maternal psychological problems during pregnancy) and child characteristics (i.e., self-regulated compliance, sex and genetic factors), for the development of externalizing problems in childhood.

## Supplementary Information

Below is the link to the electronic supplementary material.

Supplementary file1 (DOCX 42 kb)

## References

[1] Moffitt TE, Arseneault L, Belsky D, Dickson N, Hancox RJ, Harrington H (2011). A gradient of childhood self-control predicts health, wealth, and public safety. Proc Natl Acad Sci USA.

[2] Eiden RD, Edwards EP, Leonard KE (2007). A conceptual model for the development of externalizing behavior problems among kindergarten children of alcoholic families: role of parenting and children's self-regulation. Dev Psychol.

[3] Olson SL, Bates JE, Sandy JM, Lanthier R (2000). Early developmental precursors of externalizing behavior in middle childhood and adolescence. J Abnorm Child Psychol.

[4] Kochanska G, Coy KC, Murray KT (2001). The development of self-regulation in the first four years of life. Child Dev.

[5] Kochanska G, Aksan N, Koenig AL (1995). A longitudinal study of the roots of preschoolers' conscience: committed compliance and emerging internalization. Child Dev.

[6] Feldman R, Klein PS (2003). Toddlers' self-regulated compliance to mothers, caregivers, and fathers: implications for theories of socialization. Dev Psychol.

[7] Buss C, Davis EP, Hobel CJ, Sandman CA (2011). Maternal pregnancy-specific anxiety is associated with child executive function at 6–9 years age. Stress.

[8] Korja R, Nolvi S, Grant KA, McMahon C (2017). The relations between maternal prenatal anxiety or stress and child's early negative reactivity or self-regulation: a systematic review. Child Psychiatry Hum Dev.

[9] Henrichs J, Schenk JJ, Kok R, Ftitache B, Schmidt HG, Hofman A (2011). Parental family stress during pregnancy and cognitive functioning in early childhood: the generation R study. Early Child Res Q.

[10] Madigan S, Oatley H, Racine N, Fearon RMP, Schumacher L, Akbari E (2018). A meta-analysis of maternal prenatal depression and anxiety on child socioemotional development. J Am Acad Child Psychiatry.

[11] O'Connor TG, Heron J, Golding J, Glover V, Team AS (2003). Maternal antenatal anxiety and behavioural/emotional problems in children: a test of a programming hypothesis. J Child Psychol Psychiatry.

[12] Korhonen M, Luoma I, Salmelin R, Tamminen T (2014). Maternal depressive symptoms: associations with adolescents' internalizing and externalizing problems and social competence. Nord J Psychiatry.

[13] O'Connor TG, Heron J, Glover V, Team AS (2002). Antenatal anxiety predicts child behavioral/emotional problems independently of postnatal depression. J Am Acad Child Psychiatry.

[14] Van den Bergh BRH, Marcoen A (2004). High antenatal maternal anxiety is related to ADHD symptoms, externalizing problems, and anxiety in 8-and 9-year-olds. Child Dev.

[15] O'Donnell KJ, Glover V, Barker ED, O'Connor TG (2014). The persisting effect of maternal mood in pregnancy on childhood psychopathology. Dev Psychopathol.

[16] MacKinnon N, Kingsbury M, Mahedy L, Evans J, Colman I (2018). The association between prenatal stress and externalizing symptoms in childhood: evidence from the avon longitudinal study of parents and children. Biol Psychiatry.

[17] Leijten P, Gardner F, Melendez-Torres GJ, Knerr W, Overbeek G (2018). Parenting behaviors that shape child compliance: a multilevel meta-analysis. PLoS ONE.

[18] Kok R, Bakermans-Kranenburg MJ, van Ijzendoorn MH, Velders FP, Linting M, Jaddoe VW (2013). The role of maternal stress during pregnancy, maternal discipline, and child COMT Val158Met genotype in the development of compliance. Dev Psychobiol.

[19] Van den Bergh BRH, Mulder EJH, Mennes M, Glover V (2005). Antenatal maternal anxiety and stress and the neurobehavioural development of the fetus and child: links and possible mechanisms A review. Neurosci Biobehav Rev.

[20] Henrichs J, Van den Bergh BRH, Gendolla GHE, Tops M, Koole SL (2015). Perinatal developmental origins of self regulation. Handbook of biobehavioral approaches to self regulation.

[21] Bridgett DJ, Burt NM, Edwards ES, Deater-Deckard K (2015). Intergenerational transmission of self-regulation: a multidisciplinary review and integrative conceptual framework. Psychol Bull.

[22] Glover V, O'Connor TG, O'Donnell K (2010). Prenatal stress and the programming of the HPA axis. Neurosci Biobehav Rev.

[23] Lengua LJ, Zalewski M, Fisher P, Moran L (2013). Does HPA-Axis dysregulation account for the effects of income on effortful control and adjustment in preschool children?. Infant Child Dev.

[24] Scher A, Hall WA, Zaidman-Zait A, Weiinberg J (2010). Sleep quality, cortisol levels, and behavioral regulation in toddlers. Dev Psychobiol.

[25] Hart H, Radua J, Nakao T, Mataix-Cols D, Rubia K (2013). Meta-analysis of functional magnetic resonance imaging studies of inhibition and attention in attention-deficit/hyperactivity disorder exploring task-specific, stimulant medication, and age effects. JAMA Psychiatry.

[26] Fan J, McCandliss BD, Fossella J, Flombaum JI, Posner MI (2005). The activation of attentional networks. Neuroimage.

[27] Rijlaarsdam J, Stevens GWJM, Jansen PW, Ringoot AP, Jaddoe VWV, Hofman A (2014). Maternal childhood maltreatment and offspring emotional and behavioral problems: maternal and paternal mechanisms of risk transmission. Child Maltreat.

[28] Schuetze P, Eiden RD, Colder CR, Huestis MA, Leonard KE (2018). Prenatal risk and infant regulation: indirect pathways via fetal growth and maternal prenatal stress and anger. Child Dev.

[29] Field T, Diego M, Hernandez-Reif M, Salman F, Schanberg S, Kuhn C (2002). Prenatal anger effects on the fetus and neonate. J Obstet Gynaecol.

[30] Muscatello MRA, Lorusso S, Bruno A, Reale R, La Ciura G, Lagana AS (2016). Anger in women treated with assisted reproductive technology (ART): effects on mother and newborn. J Matern-Fetal Neo Med.

[31] Pappa I, St Pourcain B, Benke K, Cavadino A, Hakulinen C, Nivard MG (2016). A genome-wide approach to children's aggressive behavior: the EAGLE consortium. Am J Med Genet B.

[32] Wray NR, Lee SH, Mehta D, Vinkhuyzen AAE, Dudbridge F, Middeldorp CM (2014). Research review: polygenic methods and their application to psychiatric traits. J Child Psychol Psychiatry.

[33] Dick DM, Agrawal A, Keller MC, Adkins A, Aliev F, Monroe S (2015). Candidate gene-environment interaction research: reflections and recommendations. Perspect Psychol Sci.

[34] Kochanska G, Woodard J, Kim S, Koenig JL, Yoon JE, Barry RA (2010). Positive socialization mechanisms in secure and insecure parent-child dyads: two longitudinal studies. J Child Psychol Psychiatry.

[35] Else-Quest NM, Hyde JS, Goldsmith HH, Van Hulle CA (2006). Gender differences in temperament: a meta-analysis. Psychol Bull.

[36] Basten M, Tiemeier H, Althoff RR, van de Schoot R, Jaddoe VWV, Hofman A (2016). The stability of problem behavior across the preschool years: an empirical approach in the general population. J Abnorm Child Psychol.

[37] Smith CL, Day KL (2018). Parenting, anger, and effortful control as predictors of child externalizing behavior: the role of child sex as a moderator. Int J Behav Dev.

[38] Farrell AD, Danish SJ (1993). Peer drug associations and emotional restraint - causes or consequences of adolescents drug-use. J Consult Clin Psychol.

[39] Crockett LJ, Raffaelli M, Shen YL (2006). Linking self-regulation and risk proneness to risky sexual behavior: pathways through peer pressure and early substance use. J Res Adolesc.

[40] Olson SL, Sameroff A, Kerr DCR, Lopez NL, Wellman HM (2005). Developmental foundations of externalizing problems in young children: the role of effortful control. Dev Psychopathol.

[41] Lemery-Chalfant K, Doelger L, Goldsmith HH (2008). Genetic relations between effortful and attentional control and symptoms of psychopathology in middle childhood. Infant Child Dev.

[42] Pappa I, Fedko IO, Mileva-Seitz VR, Hottenga JJ, Bakermans-Kranenburg MJ, Bartels M (2015). Single nucleotide polymorphism heritability of behavior problems in childhood: genome-wide complex trait analysis. J Am Acad Child Psychiatry.

[43] Luijk MPCM, Saridjan N, Tharner A, van Ijzendoorn MH, Bakermans-Kranenburg MJ, Jaddoe VWV (2010). Attachment, depression, and cortisol: deviant patterns in insecure-resistant and disorganized infants. Dev Psychobiol.

[44] Tharner A, Luijk MPCM, van IJzendoorn MH, Bakermans-Kranenburg MJ, Jaddoe VWV, Hofman A (2012). Infant attachment, parenting stress, and child emotional and behavioral problems at age 3 years. Parent-Sci Pract.

[45] Kooijman MN, Kruithof CJ, van Duijn CM, Duijts L, van FrancoIJzendoorn OHMH (2016). The generation R study: design and cohort update 2017. Eur J Epidemiol.

[46] Tiemeier H, Velders FP, Szekely E, Roza SJ, Dieleman G, Jaddoe VW (2012). The generation R study: a review of design, findings to date, and a study of the 5-HTTLPR by environmental interaction from fetal life onward. J Am Acad Child Adolesc Psychiatry.

[47] De Beurs E (2004) BSI: Brief Symptom Inventory. Handleiding [manual]. (Leiden: Psychologische Instrumenten Test en Services, B.V. (PITS).)

[48] Derogatis LR, Melisaratos N (1983). The brief symptom inventory: an introductory report. Psychol Med.

[49] Kochanska G, Aksan N (1995). Mother-child mutually positive affect, the quality of child compliance to requests and prohibitions, and maternal control as correlates of early internalization. Child Dev.

[50] Kuczynski L, Kochanska G, Radke-Yarrow M, Girnius-Brown O (1987). A developmental interpretation of young childrens noncompliance. Dev Psychol.

[51] Linting M, Meulman JJ, Groenen PJF, van der Kooij AJ (2007). Nonlinear principal components analysis: introduction and application. Psychol Methods.

[52] Achenbach TM, Rescorla LA (2000) Manual for ASEBA Preschool Forms & Profiles (Research Center for Children, Youth, & Families, Burlington: University of Vermont)

[53] Achenbach TM, Rescorla LA (2001) Manual for ASEBA School-Age Forms & Profiles. (Research Center for Children, Youth, & Families., Burlington: University of Vermont)

[54] Medina-Gomez C, Felix JF, Estrada K, Peters MJ, Herrera L, Kruithof CJ (2015). Challenges in conducting genome-wide association studies in highly admixed multi-ethnic populations: the Generation R Study. Eur J Epidemiol.

[55] Choi SW, O'Reilly PF (2019). PRSice-2: Polygenic Risk Score software for biobank-scale data. Gigascience.

[56] Delucchi KL, Bostrom A (2004). Methods for analysis of skewed data distributions in psychiatric clinical studies: working with many zero values. Am J Psychiatry.

[57] Hayes AF (2013). Introduction to mediation, moderation, and conditional process analysis: a regression-based approach.

[58] Dirks MA, De Los RA, Briggs-Gowan M, Cella D, Wakschlag LS (2012). Annual research review: embracing not erasing contextual variability in children's behavior - theory and utility in the selection and use of methods and informants in developmental psychopathology. J Child Psychol Psychiatry.

[59] Loomans EM, van der Stelt O, van Eijsden M, Gemke RJBJ, Vrijkotte T, Van den Bergh BRH (2011). Antenatal maternal anxiety is associated with problem behaviour at age five. Early Human Dev.

[60] Chi TC, Hinshaw SP (2002). Mother-child relationships of children with ADHD: the role of maternal depressive symptoms and depression-related distortions. J Abnorm Child Psychol.

[61] Elgar FJ, Mills RS, McGrath PJ, Waschbusch DA, Brownridge DA (2007). Maternal and paternal depressive symptoms and child maladjustment: the mediating role of parental behavior. J Abnorm Child Psychol.

[62] Taraban L, Shaw DS, Leve LD, Natsuaki MN, Ganiban JM, Reiss D (2019). Parental depression, overreactive parenting, and early childhood externalizing problems: moderation by social support. Child Dev.

[63] Totsika V, Hastings RP, Emerson E, Hatton C (2019). Early years parenting mediates early adversity effects on problem behaviors in intellectual disability. Child Dev.

[64] Velders FP, Dieleman G, Henrichs J, Jaddoe VWV, Hofman A, Verhulst FC (2011). Prenatal and postnatal psychological symptoms of parents and family functioning: the impact on child emotional and behavioural problems. Eur Child Adolesc Psychiatry.

[65] Smith TW, Glazer K, Ruiz JM, Gallo LC (2004). Hostility, anger, aggressiveness, and coronary heart disease: an interpersonal perspective on personality, emotion, and health. J Pers.

[66] Abbott PW, Gumusoglu SB, Bittle J, Beversdorf DQ, Stevens HE (2018). Prenatal stress and genetic risk: how prenatal stress interacts with genetics to alter risk for psychiatric illness. Psychoneuroendocrinology.

[67] Taraban L, Shaw DS, Leve LD, Wilson MN, Dishion TJ, Natsuaki MN (2017). Maternal depression and parenting in early childhood: contextual influence of marital quality and social support in two samples. Dev Psychol.

[68] Kochanska G, Kim S, Boldt LJ (2013). Origins of children's externalizing behavior problems in low-income families: toddlers' willing stance toward their mothers as the missing link. Dev Psychopathol.

[69] Rhee SH, Friedman NP, Watts AKS, Corley RP, Hewitt JK, Robinson J (2018). The association between toddlerhood self-control and later externalizing problems. Behav Genet.

[70] Barnes JC, Boutwell BB, Beaver KM, Gibson CL (2013). Analyzing the origins of childhood externalizing behavioral problems. Dev Psychol.

[71] Jansen PR, Polderman TJC, Bolhuis K, van der Ende J, Jaddoe VWV, Verhulst FC (2018). Polygenic scores for schizophrenia and educational attainment are associated with behavioural problems in early childhood in the general population. J Child Psychol Psychiatry.

[72] Bulik-Sullivan B, Finucane HK, Anttila V, Gusev A, Day FR, Loh PR (2015). An atlas of genetic correlations across human diseases and traits. Nat Genet.

[73] Elam KK, Clifford S, Shaw DS, Wilson MN, Lemery-Chalfant K (2019). Gene set enrichment analysis to create polygenic scores: a developmental examination of aggression. Transl Psychiatry.

[74] Eisenberg N, Spinrad TL, Fabes RA, Reiser M, Cumberland A, Shepard SA (2004). The relations of effortful control and impulsivity to children's resiliency and adjustment. Child Dev.

