# Supplementary material for: Maternal Psychological Problems During Pregnancy and Child Externalizing Problems: Moderated Mediation Model with Child Self-regulated Compliance and Polygenic Risk Scores for Aggression
Source: Child Psychiatry Hum Dev. 2021 Mar 20;53(4):654–66. doi: 10.1007/s10578-021-01154-1 (PMC9287202; doi:10.1007/s10578-021-01154-1)
Supplement: Supplementary file 1 — Supplementary file1 (DOCX 42 kb) [file 10578_2021_1154_MOESM1_ESM.docx]

# Supplement 1. Genotype quality control and imputation

Quality control steps were performed on the genotype data in PLINK 2.0 [1], included filtering variants for minor allele frequency (MAF < 0.01), Hardy–Weinberg disequilibrium (*p* < 0.000001), and missing rate (> 0.05). Genotype data that passed quality control were subsequently imputed using the Haplotype Reference Consortium (HRC) release 1.1 as the reference panel. In total, 5,319,024 variants were available for calculating polygenic risk scores.

Excluded (*n* = 165) because of missing data on aggression PRSs.

*N* = 522 for moderated mediation analyses

Excluded (*n* =107) because of missing data on aggression PRSs.

*N* = 353 for moderated mediation analyses

Mothers with available data on maternal psychological problems during pregnancy *N*=750

Excluded (*n* = 290) because of missing data on teacher reports of externalizing problems.

Final Study Sample: *N* = 460

Excluded (*n* = 63) because of missing data on mother reports of externalizing problems:

Final Study Sample: *N* = 687

Children with compliance data available (at least one out of two tasks)

*N* = 821

Excluded (*n* =50) because of missing data on maternal psychological problems during pregnancy

Excluded (*n* = 21) because of paired data

Mother-child dyad without paired data *N* = 800

Excluded (*n* = 31) because of

technical or procedural difficulties

Families participating in Focus Cohort lab visit at age 3

*N* = 852

**Fig. S1** Flowchart of the study population.

**Table S1 Sensitivity analysis**: maternal psychological problems during pregnancy predicting child externalizing problems, mediated by self-regulated compliance, adjusting for maternal psychological problems at child age 3.

| Paths | Externalizing problems | | | | |  |
| --- | --- | --- | --- | --- | --- | --- |
|  | Mother reports ( *N* = 640) | |  | Teacher reports ( *N* = 415) | | |
|  | *β* (SE) | 95% CI |  | *β* (SE) | 95% CI | |
| Maternal affective problems during pregnancy → Self-regulated compliance at age 3 | **-**.11 (.05) | **-.211, -.018** |  | **-**.18 (.07) | **-.318, -.050** | |
| Maternal affective problems at age 3 → Self-regulated compliance at age 3 | **-**.001 (.04) | **-**.085, .084 |  | .01 (.05) | **-**.100, .113 | |
| Self-regulated compliance at age 3→ Externalizing problems at age 6 | **-**.12 (.04) | **-.194, -.044** |  | **-**.09 (.05) | -.189, .000 | |
| Maternal affective problems at age 3 → Externalizing problems at age 6 | .17 (.04) | **.091, .254** |  | .14 (.05) | **.037, .244** | |
| Maternal affective problems during pregnancy (direct effect) | .09 (.05) | **-**.007, .180 |  | **-**.06 (.07) | **-**.187, .076 | |
| Indirect effect via self-regulated compliance | .01 (.01) | **.003, .035** |  | .02 (.01) | **.002, .052** | |
|  | | | | | |  |
| Maternal hostility during pregnancy → Self-regulated compliance at age 3 | **-**.08 (.05) | *-*.178, .009 |  | **-**.13 (.07) | -.261, .000 | |
| Maternal hostility at age 3 → Self-regulated compliance at age 3 | **-**.03 (.04) | **-**.114, .048 |  | **-**.06 (.05) | **-**.157, .046 | |
| Self-regulated compliance at age 3→ Externalizing problems at age 6 | **-**.12 (.04) | **-.194, -.045** |  | **-**.09 (.05) | **-**.182,   .007 | |
| Maternal hostility at age 3 → Externalizing problems at age 6 | .26 (.04) | .**179, .334** |  | .13 (.05) | .**029, .226** | |
| Maternal hostility during pregnancy (direct effect) | **-**.002 (.05) | **-**.089, .089 |  | -.03 (.07) | **-**.160, . 095 | |
| Indirect effect via Self-regulated compliance at age 3 | .01 (.01) | **-**.001, .029 |  | .01 (.01) | **-**.001, .041 | |

All covariates are included in the model as described in Methods section

Paths significant as indicated by the confidence intervals are presented in bold

*β*, standardized coefficient; SE, standardized error

**Table S2** Regression analysis for polygenic risk scores and externalizing problems at each p-value thresholds. Results are corrected for child gender and age at the assessment of outcome.

|  | - Polygenic Risk Score for Aggression | | | | |  |
| --- | --- | --- | --- | --- | --- | --- |
| - Outcome: - Externalizing problems | - P-value Threshold | - *β* | - SE | - *p* | - *R*^2^ | |
| Mother reports ^a^ | - .01 | - .052 | - .043 | - .223 | - .003 | |
|  | - .1 | - **.101** | - **.043** | - **.019** | - **.010** | |
|  | - 1 | - .039 | - .043 | - .362 | - .002 | |
| - Teacher reports ^b^ | - .01 | - .018 | - .053 | - .731 | - 3.24×10^−4^ | |
|  | - .1 | - .054 | - .053 | - .307 | - .003 | |
|  | - 1 | - -.027 | - .053 | - .606 | - .001 | |

^a^  *N* = 522; ^b^ *N* = 353

*β*, standardized coefficient; SE, standardized error

**Reference**

1. Purcell S*, et al.* (2007) PLINK: A tool set for whole-genome association and population-based linkage analyses. *American Journal of Human Genetics* 81:559-575
